# The Promise of 3D Biomaterial Bioprinting for Wound-Healing and Skin Tissue Restoration

**DOI:** 10.3390/life16050718

**Published:** 2026-04-23

**Authors:** Moatter B. Syed, Tamer A. E. Ahmed, Maxwell T. Hincke

**Affiliations:** 1 Department of Cellular and Molecular Medicine, Faculty of Medicine, University of Ottawa, Ottawa, ON K1H 8M5, Canada; msyed014@uottawa.ca (M.B.S.); tahmed@uottawa.ca (T.A.E.A.); 2 Department of Innovation in Medical Education, Faculty of Medicine, University of Ottawa, Ottawa, ON K1H 8M5, Canada

**Keywords:** skin, 3D bioprinting, natural biomaterials, skin wound-healing, hydrogels, bioinks

## Abstract

Wound-healing and skin regeneration are the focus of intensive research, driven by a rapidly expanding global market and the growing clinical demand for more effective interventions engineered to actively direct and enhance tissue regeneration. Recent advances in biomaterial engineering and 3D bioprinting have accelerated the development of highly customized, functional constructs mimicking native tissue. Together, these innovations are reshaping therapeutic strategies and expanding the translational potential of next-generation skin substitutes. This review presents an overview of the evolution of material printing technologies and the different categories of 3D bioprinting techniques and processing methods, followed by an evaluation of the properties of natural biomaterials as bioinks for skin wound-healing and their application in skin tissue engineering. Moreover, we provide a comprehensive global market analysis, with consideration of costs, benefits, and a SWOT analysis to identify the full potential of this technology for the development of novel skin wound-healing products. Recommendations and future perspectives are provided to guide researchers, clinicians, and industry partners on the current state and potential of adopting 3D bioprinting with natural biomaterials for effective wound-healing therapies.

## 1. Introduction

**Figure 1:**
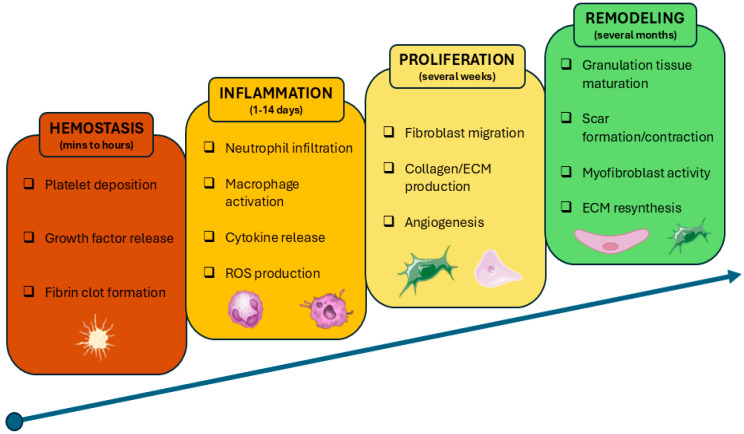
Overview of the four overlapping phases of skin wound-healing. wound-healing is a dynamic, regulated process that occurs in four overlapping but distinct phases: hemostasis, inflammation, proliferation, and remodeling. Each phase involves specific events, key cell types, and biomarkers that coordinate to restore skin integrity and function, thereby contributing to tissue repair and wound-healing over time.

As the body’s largest and most functionally intricate organ, the skin provides essential barriers and homeostatic functions, and any disruption of its integrity leads to tightly coordinated phases of wound-healing: hemostasis, inflammation, proliferation, and remodeling [1,2] (Figure 1). Skin wounds affect millions of people worldwide and can lead to severe disability and reduced quality of life [3]. Current treatments, such as skin transplantation and autografting, are limited by donor site availability, the risk of infection, and impaired healing [4]. Chronic skin wounds—including diabetic foot and venous leg ulcers—affect 1–2% of the population in developed countries and contribute to an annual global healthcare burden exceeding USD 250 billion [5,6,7]. This rising demand, driven by aging populations and the prevalence of chronic diseases, underscores the need for cost-effective and advanced wound care strategies. Tissue engineering strategies, including the development of cell-laden hydrogels and bioactive scaffolds aimed at restoring the structure and function of skin, have culminated in the emergence of 3D bioprinting as an effective treatment option.

**Figure 2:**
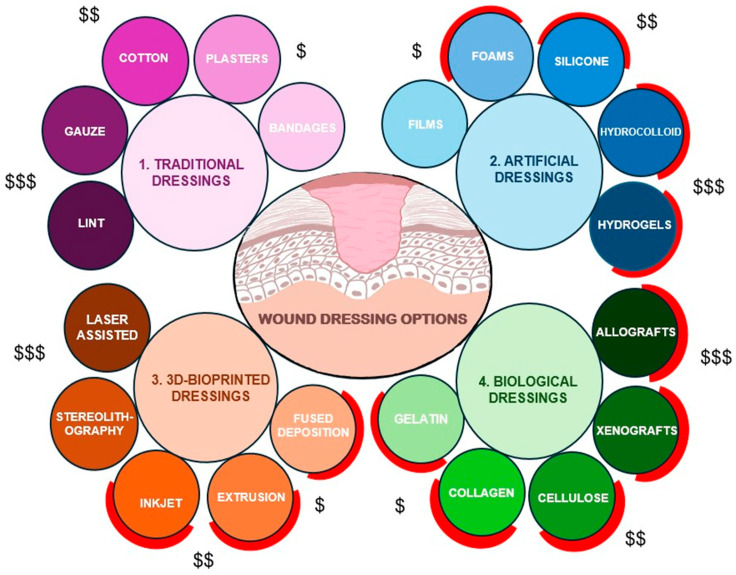
**Classification of skin wound dressing options for the treatment of acute and chronic wounds.** This illustration shows four main categories of dressings: (1) traditional dressings (e.g., gauze, bandages, plasters, lint, and cotton wool); (2) artificial dressings (e.g., foams, hydrogels, hydrocolloids, and films); (3) 3D bioprinted dressings developed using different bioprinting technologies (e.g., fused deposition modeling, extrusion-based bioprinting, inkjet bioprinting, stereolithography, and laser-assisted bioprinting); and (4) biological dressings (e.g., collagen, gelatin, cellulose, allografts, and xenografts). Within each category, relative cost is indicated using dollar-sign notation ($, $$, $$$), where increasing dollar signs denote greater expense; darker-shaded circles likewise represent relatively more expensive options, whereas lighter-shaded circles represent less expensive options. The outer red shading denotes dressing types primarily targeted for treating chronic wounds [9,10,11,12].

Various wound dressings (Figure 2) are used clinically, ranging from traditional gauze and bandages to artificial, biological, and bioactive formulations, with newer materials designed to retain moisture, prevent infection, and actively stimulate regeneration. Some key tissue engineering strategies in cutaneous wound-healing help replicate skin tissue by utilizing scaffolds, cellular therapies, and growth factors. Scaffolds can reinforce an ECM-like environment that supports cell infiltration and proliferation. Cellular components, including keratinocytes, fibroblasts, and mesenchymal stem cells, contribute to epidermal and dermal skin regeneration. Meanwhile, signaling molecules such as vascular endothelial growth factor, epidermal growth factor, and transforming growth factor-β help regulate inflammation, granulation, and remodeling. However, to overcome the limitations of conventional wound therapies, 3D bioprinting has emerged as a promising alternative for treating injured skin and facilitating wound-healing. By utilizing computer scanning and advanced imaging, 3D bioprinting can rapidly produce skin grafts tailored to irregular, deep wounds, ensuring high throughput and reproducibility. The layer-by-layer deposition method enables the direct printing of skin tissue onto wound sites, promoting seamless integration with surrounding tissues [8].

Despite advancements in skin tissue engineering, several challenges remain, including the restoration of skin pigmentation, wound contraction, limited dermal elasticity, long-term scarring, incomplete nerve and appendage regeneration, as well as insufficient vasculature to promote tissue viability [8,13,14]. To address such challenges, current research is focusing on standardizing bioinks that incorporate growth factors, mesenchymal stem cells, and multifunctional hydrogels, which can uniquely adapt to the wound environment and induce tissue remodeling [8]. As research continues to refine 3D bioprinting for skin tissue engineering, addressing these specific challenges is crucial in developing functional skin grafts that not only restore the skin’s structural integrity but also enhance physiological and sensory functions, thereby improving patient outcomes and overall quality of life. Although a number of reviews have addressed 3D bioprinting in skin tissue engineering and wound-healing, these are often centered on specific aspects of the field, such as printing modalities, scaffold fabrication, or bioink composition. The novelty of the present review lies in its broader integrative approach, which combines the discussion of bioprinting technologies, natural biomaterials, complementary strategies, wound-healing functionality, and translational market considerations within a single framework. This structure is intended to provide readers with a more complete understanding of how material properties, fabrication approaches, and clinical needs intersect in the development of bioprinted skin substitutes.

This narrative review was informed by a targeted literature search conducted using PubMed, Scopus, and Google Scholar. Search terms included combinations of “3D bioprinting,” “wound-healing,” “skin regeneration,” “bioinks,” “hydrogels,” “eggshell membrane,” “natural biomaterials,” and “skin wound-healing”. The search primarily focused on studies published from 2015 onwards, with additional seminal articles included where relevant. Studies were selected based on their relevance to the scope of this review, particularly those addressing biomaterials, fabrication strategies, and emerging approaches for skin tissue engineering and wound-healing.

## 2. Evolution of 3D Bioprinting Techniques

Conventional printing technologies, such as inkjet, extrusion, or laser-based methods, have been primarily developed for the deposition of non-living materials (e.g., polymers or metals) onto substrates in a two-dimensional (2D) structure [15,16]. Such printing systems are optimized for precision, resolution, and speed to help produce structural elements that require no biological or cellular components. Conversely, bioprinting is an advanced method of additive manufacturing that integrates biological components, such as living cells, biomaterials, growth factors, and other bioactive molecules, into a three-dimensional (3D) construct with the primary target of replicating native tissue structure and function [17]. More importantly, the bioinks used for bioprinting must meet specific criteria for biocompatibility, sterility, mechanical and rheological integrity, printability, and cell viability. Conventional 3D printing prioritizes geometrics and mechanics, whereas bioprinting supports cellular behavior, tissue maturation, and long-term functioning, allowing its use for various applications [18]. Importantly, this transition from conventional printing to bioprinting was not simply a change in printable materials, but a shift in the performance criteria. In bioprinting, the printed construct must not only retain its intended structure but also preserve the viability of living cells and support tissue maturation [18].

In this context, material selection and printing strategy are restricted by the interplay between printability, biocompatibility, and biological function. Printability refers to the ability of a bioink to be reproducibly dispensed and patterned into a stable structure with acceptable shape fidelity, resolution, and layer stacking, while minimizing nozzle clogging, spreading, filament collapse, or postprinting deformation. This depends on the rheological and physicochemical parameters such as viscosity, shear-thinning behavior, viscoelasticity, gelation kinetics, and crosslinking. Such properties must be balanced against biological requirements, including cell viability, cytocompatibility within crosslinking conditions, degradation behavior, matrix support for cell adhesion, proliferation, and differentiation [18]. Thus, the central challenge in bioprinting is not simply if the material can be printed, but whether it can be printed without compromising cellular performance or long-term performance.

### 2.1. Evolution of Material Printing Technology and Different Categories

**Figure 3:**
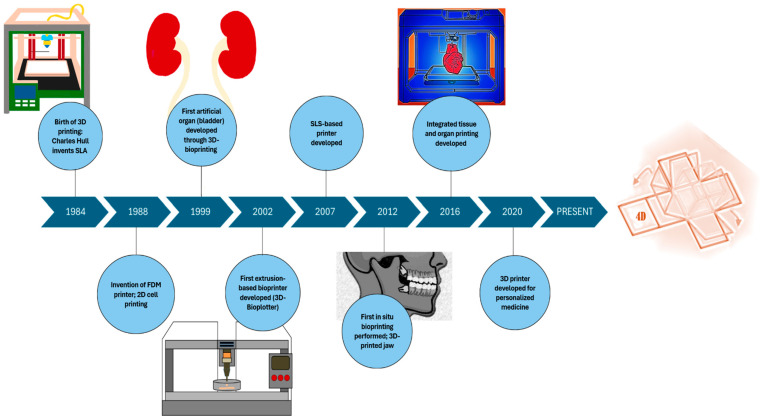
**Evolution of significant breakthroughs in 3D bioprinting technologies.** A historical timeline illustrating the major milestones in the development of 3D bioprinting technologies [19,20,21]. The origins of 3D printing date back to 1984, when Charles Hull invented stereolithography, which enabled the layer-by-layer construction of 3D structures [21]. The foundational phase of 3D printing focused on fabricating medical models and developing printing guidelines. Subsequent milestones include cell-based printing, tissue-engineered organ constructs (e.g., bladder models), and the introduction of extrusion-based printing. More recent advances include integrated tissue and organ printing, in situ bioprinting (e.g., craniofacial bone construction), and the emergence of bioprinting for personalized, patient-specific applications.

The development of material printing technologies has progressed over the last four decades, from traditional 2D techniques to more advanced 3D techniques, to formulate materials ranging from simple to complex for biomedical applications (Figure 3). Additive manufacturing (AM), also known as three-dimensional (3D) printing, is regarded as the most advanced process of material printing, where layers are built in three dimensions. The evolution of these technologies reflects a progressive effort to overcome the mismatch between printing performance and biological computability. Early additive manufacturing platforms were highly effective in shaping solid materials, but were not designed to process hydrated, soft, cell-laden formulations. As the field advanced toward biomedical applications, technological development was increasingly driven by the need to control structural integrity, as well as cellular response. This led to the emergence of multiple additive manufacturing categories, each with distinct trade-offs in resolution, printable viscosity range, mechanical limits, processing conditions, and cell compatibility. As such, no single printing modality is universally optimal but rather they are dependent on assigned priority (i.e., structure, printing speed, precision, etc.)

#### 2.1.1. Early Limitations and Material Challenges

First developed in the early 1980s, synthetic materials such as photocurable resins and polymethyl methacrylate (PMMA) lacked biocompatibility and were unsuitable for disease management or organ transplantation [22]. Subsequently, 3D printing advanced to utilize biocompatible and nondegradable materials, as well as synthetic biodegradable polymers such as polylactic acid, polycaprolactone, polyglycolic acid, and polyvinyl alcohol [23,24]. Although these materials improved degradability and expanded biomedical utility, specifically for scaffolds and implants, many still required harsh processing conditions, including high temperature or organic solvents, further restricting cell incorporation [22,24]. Such materials have been complemented by natural biomaterials, such as collagen, gelatin, alginate, fibrin, and hyaluronic acid, or by composite bioinks to provide further improved cell adhesion and biological function [22]. This stage enabled the development of medical implants, including the successful construction of a 3D-bioprinted bladder using a collagen-based polymeric scaffold seeded with autologous cells [25] and a 3D-bioprinted trachea from methacrylated gelatin (GelMA), chondroitin sulfate methacryloyl, and elastin methacryloyl composite hydrogels [26]. These advances strengthened the relationship between material properties, printability conditions, and functional tissue criteria.

#### 2.1.2. Innovations in Cell-Laden Hydrogels and Composite Bioinks

Progressing further, 3D printing shifted towards cell-laden hydrogels and composite bioinks, combining structural integrity with biological function. This moved bioprinting beyond passive scaffolds and toward the development of living constructs that support cell survival and regenerative activity. Hydrogels derived from materials such as GelMA, methacrylated hyaluronic acid (HAMA), and alginate–methylcellulose composites were further optimized for improved rheological properties and crosslinking mechanisms [27]. These materials were important as they provided a favorable environment for encapsulated cells, while also allowing control over printability through viscosity tuning, shear-thinning behavior, and gelation kinetics [27]. These innovations enabled the printing of more complex 3D tissue constructs for skin regeneration [22]. However, this stage highlighted a key challenge in bioink development, where materials that support cell signaling often exhibit poor mechanical strength or limited shape fidelity. Whereas formulations with improved shape fidelity may expose cells to greater shear stress during deposition. The integration of nanomaterials, such as graphene oxide, bioactive glass, or ceramic nanoparticles, enhanced the mechanical strength and print fidelity of constructs for skin tissue engineering [28,29]. Another key milestone from this stage was the demonstration of more structurally complex constructs, including in situ bioprinting of a functional, patient-specific jaw using composite bioinks [30], showcasing the wide scope of 3D bioprinting. Furthermore, these developments showed that progress in the field is not only dependent on new biomaterial compositions but also on the ability to engineer composite systems that balance printability, mechanical support, and bioactivity.

#### 2.1.3. Current Advances: Smart Biomaterials and 4D Bioprinting

The most groundbreaking and current stage of 3D bioprinting involves the formation of biologically active, patient-specific structures. At this stage, the field has moved beyond recreating tissue geometry and is focused on producing constructs that interact with the wound microenvironment. This includes the addition of decellularized extracellular matrix (dECM)-based bioinks, which provide tissue-specific biochemical cues, and the development of “smart” biomaterials such as thermo-responsive, pH-responsive, and enzyme-responsive hydrogels [31,32] to facilitate wound-healing and restore other biological functions. These materials are paving the way for 4D bioprinting, in which printed constructs can change over time in response to specific physiological conditions [33], including fluctuations in moisture, pH, enzyme activity, inflammation, or tissue remodeling. Smart biomaterials, therefore, offer the possibility of more responsive and personalized wound care. However, the development of such advanced systems also introduces unresolved challenges, including difficulty with the precise control of time-dependent material activity and maintaining structural stability with dynamic changes.

#### 2.1.4. Overview and Progression of 3D Printing Technologies

Based on the physical state of the starting material, additive manufacturing processes can be divided into four distinct categories: liquid, filament/paste, powder, and solid sheet material formation [34]. Different types of Three-dimensional printing have emerged within the past two decades, including DLP (digital light processing), FDM (fused deposition modeling), SLS (selective laser sintering), SLM (selective laser melting), 3D inkjet printing/binder jetting, and LOM (laminated object manufacturing) [35]. DLP is a biomaterial fabrication technique that utilizes light or radiation to form high-resolution and complex 3D structures at both micro- and nanoscale [36]. It offers precise geometric patterning of biological materials and recapitulates the functionality of native tissues. FDM is an extrusion-based AM technology, which involves melting raw material to build 3D shapes [37]. SLS develops 3D structures by selectively fusing powder beds layer by layer using a high-power laser or electronic beam [38]. Like SLS, selective laser melting uses a similar layer deposition method; however, in SLM, the material is in a molten state, and the melting process is modified to liquefy the powder using a laser, which is then cooled to form a solid [39]. 3D inkjet printing involves printing a liquid binder onto a powder bed or jetting a photopolymer to form a 2D pattern, which is then stacked layer by layer to build a fully formed structure [40]. Laminated object manufacturing constructs different parts using thin sheets of film, including paper, plastic, fibers, metals, or composites. Layered sheets are cut using a laser beam and bonded together [35].

There has been significant progress in 3D bioprinting techniques, advancing the precise and functional deposition of biomaterials for wound-healing applications. Among the most widely used techniques, extrusion-based bioprinting has become the primary mode for depositing highly viscous, cell-laden bioinks with improved structural integrity and cell viability [41]. Advanced extrusion-based systems feature multi-nozzle platforms, temperature-controlled printing beds and chambers, as well as fine-tuned pneumatic dispensing, allowing layer-by-layer deposition of full-thickness constructs [42,43]. Inkjet bioprinting, which has traditionally been limited by low-viscosity constraints, has been optimized using piezoelectric and thermal actuation for higher-throughput cell deposition [44], which is a critical step for printing growth factors or cell components. Laser-assisted bioprinting, which utilizes laser energy to transfer biomaterials onto substrates, has been enhanced to achieve micron-level resolution and minimal shear stress during printing, making it an ideal choice for formulating fragile cell types [45,46]. More recently, DLP and SLA have enabled the photo-polymerization of light-sensitive bioinks into more complex shapes with improved precision in reproducing native skin structures and layers [47]. Additionally, situ bioprinting techniques have revolutionized skin regeneration by enabling the direct deposition of biomaterials and cells onto wound sites using handheld or robotically operated devices. Such real-time printing systems incorporate the imaging and mapping of wounds to customize the 3D constructs to the patient’s skin topography and condition [48,49].

The processing of biomaterials is a crucial stage that necessitates standardized methodologies to develop biocompatible scaffolds for tissue engineering applications. This ensures that biomaterials are safe for clinical use and do not cause adverse reactions in humans [50]. Over the last decade, three-dimensional bioprinting has been validated as an attractive advanced additive manufacturing method in regenerative medicine and tissue engineering, enabling the development of complex tissue and organ constructs incorporating bioactive molecules [51]. Three-dimensional bioprinting has enabled the processing of biocompatible materials and cells into functional living tissues [52]. Three-dimensional bioprinting combines the features of traditional 3D printing and uses bioinks compatible with living organisms. A range of 3D printing methods and processes has been developed, including inkjet, droplet-based, extrusion-based, laser-assisted, FDM, and SLA-based printing [52]. Thus, in this context, it is essential to examine various printing technologies and their corresponding applications (Table 1).

### 2.2. Three-Dimensional Bioprinting of Biomaterials and Development of Various Bioinks for Wound-Healing

More recently, Three-dimensional bioprinting has emerged as a novel technique for applications in drug delivery, organ replacement, and regenerative medicine. 3D bioprinting serves as a promising biofabrication technology for developing advanced constructs that promote wound-healing [58]. The three main 3D bioprinting modalities used in biomedical research include laser-assisted, inkjet, and extrusion-based bioprinting [62]. Bioinks are a crucial component of these different printing methods for fabricating 3D constructs. Diverse types of natural and synthetic biomaterials have been formulated as bioinks for specific tissues, cell types, and bioprinters. Bioinks are formulations that hold cells when tissues are bioprinted, providing a solid structure where cells can survive and multiply. It refers to a printable, pre-gel, or pre-crosslinked formulation that contains living cells, hydrogels (such as alginate, collagen, or GelMA), or biological components. Various materials and combinations of different materials are used to develop these unique bioinks, including polymers, elastomers, ceramics, carbon compounds, composites, and hydrogels [63,64]. Once a bioink has been deposited and solidified through crosslinking or gelation, it is referred to as a bioprinted construct or scaffold.

#### 2.2.1. Characteristics of Biomaterials Utilized for the Development of Bioinks

Ideal bioinks for 3D printing purposes should be readily printable, biocompatible, stable, flexible, and have modifiable degradation rates [64]. Additionally, combining two or more materials is a common strategy to harness the benefits of each component [65]. Bioinks typically comprise nontoxic components and are optimized for printing temperatures that are compatible with cell survival [62]. Polymeric materials such as bioinks are biocompatible, cost-effective, and easily degradable. As such, collagen, hyaluronic acid, gelatin, sodium alginate, and chitosan are promising candidates for use as polymeric bioinks in 3D bioprinting. Typically, liquid or viscous gel bioinks are transformed into hydrogels after exposure to a crosslinking agent after printing. Additionally, many groups employ pre-crosslinking, which is a partial gelation method applied prior to or during extrusion. This is done to temporarily increase viscosity and improve shape fidelity without increasing polymer content. In alginate-based bioinks, this usually involves ionic gelation with low concentrations of Ca^2+^ salts, followed by final post-crosslinking [66]. Pre-crosslinking strategies with alginate are compatible with cell viability and exhibit viscoelastic properties suitable for extrusion bioprinting [67]. Hydrogels are commonly used in bioprinting cells due to their biocompatibility and resemblance to the extracellular matrix of tissues [68], providing a beneficial environment for cell growth. Biomaterials are typically selected based on the intended application of the end product being constructed [64,69]. For example, biomaterials such as ceramics or hard polymers used in orthodontic and orthopedic applications are mechanically stiff and have slower degradation rates, whereas natural biomaterials, such as hydrogels, which are commonly used for tissue and skin regeneration, tend to be flexible and have shorter degradation rates [64]. Effective bioinks for printing must possess optimal rheological, biological, and crosslinking properties to hold structure, as well as adequate cytocompatibility to ensure that cells in the bioink remain viable [67]. Fine-tuning of rheological properties, such as extrusion rate, viscosity, and shear stress, is also imperative for the functionality and performance of these bioinks during printing.

In alginate-based bioinks, the ratio of β-D-mannuronic acid (M) to α-L-guluronic acid (G), commonly referred to as the M:G ratio, is a key determinant of the physicochemical and biological performance of this material for 3D bioprinting. The M:G composition directly influences gelation kinetics, mechanical integrity, and print fidelity. Alginates with a high G content (low M:G) exhibit stronger and faster ionic crosslinking in the presence of divalent cations [70]. This results in highly stiff hydrogels with better shape retention and print fidelity [71]. In contrast, alginates with a high M content (high M:G ratio) will form softer, more permeable gels due to weaker crosslinking [72]. This can reduce the mechanical strength and stability of constructs; however, this composition exhibits better shear-thinning behavior and flow under extrusion, with improved biocompatibility [73,74,75]. Similar structure–function relationships are also observed in collagen-based bioinks, where collagen source and processing, as well as the pH, temperature, and ionic strength of mediums, greatly influence gelation behavior, mechanical integrity, print fidelity, and biological responses in a manner analogous to the M:G-dependent properties of alginate bioinks [76,77]. Type I collagen accounts for one-third of the total protein content in mammals and serves as the primary structural component of the ECM. Thus, because of its biocompatibility, type I is widely used in tissue engineering scaffold design and wound repair studies [77]. In particular, the α1/α2 chain ratio of telopeptide-containing collagens influences gelation, where a higher α1/α2 ratio results in slower gelation and a lower α1/α2 ratio results in faster gelation [77]. Such characteristics are advantageous for soft tissue engineering applications where cellular viability and function are critical. The optimal bioink formulation requires a trade-off between these properties or the incorporation of additional polymers, depending on the specific application.

Thus, the selection of biomaterials for the development of bioinks must be considered not only in terms of composition, but also in relation to how their mechanical, rheological, degradation, and biological properties affect scaffold performance and therapeutic outcomes in wound-healing applications.

#### 2.2.2. Advances in Bioinks Used in 3D Bioprinting Applications for Wound-Healing

Recent advances in 3D bioprinting have significantly influenced the development of novel bioink formulations for wound-healing. A primary focus in this domain has been the enhancement of bioinks with hydrogel-based materials that can morph into structurally and biologically sound scaffolds. Traditional natural polymers, such as alginate and gelatin, have been further enhanced through chemical modifications to improve printability, mechanical strength, and cell viability.

Bioinks are a central aspect of effective 3D bioprinting, as they provide a foundation for supporting cells and maintaining structural fidelity before and after the printing process. Recent studies in bioink formulation have focused on improving printability, mechanical integrity, and biocompatibility, all of which are essential for promoting effective wound-healing and skin regeneration. Hydrogels derived from natural polymers, such as alginate, gelatin, collagen, and fibrin, have been extensively studied due to their unique ECM-mimicking properties [62]. Composite formulations, such as GelMA combined with natural polymers, have demonstrated better mechanical properties and bioactivity. GelMA–chitosan composite bioinks seeded with human dermal fibroblasts and keratinocytes support bilayer skin formation and faster re-epithelialization in full-thickness wounds [78]. Another major advancement has been the formulation of skin-derived dECM bioinks, which contain biochemical signals and growth factors that promote tissue-specific cell interactions [79]. These bioinks provide a biomimetic environment that further enhances cell proliferation and ECM deposition. Some studies have demonstrated that bioprinting using skin-derived dECM-based bioinks results in improved collagen remodeling and the stratification of the epidermal layers in vivo. For example, a dECM bioink composed of porcine skin promoted the formation of full-thickness skin with vascularization and dermal regeneration [80].

##### Smart Bioinks

Novel bioinks are also being modified to respond to physiological stimuli, such as pH, temperature, or reactive oxygen species, which are important characteristics to monitor within wound environments. These “smart” bioinks enable controlled release of drugs or growth factors at the targeted site, thereby promoting anti-inflammatory effects and modulation of degradation [81]. A recent study utilized a poloxamer-based thermo-responsive hydrogel loaded with bacterial lactoferricin nanovesicles as an advanced wound dressing to manage chronic wound infections [82], highlighting the potential of drug-loaded hydrogels for accelerated wound-healing, anti-inflammatory activity, and antibacterial effects. Another valuable approach to formulating effective bioinks for wound-healing is co-printing multiple cell types, such as dermal fibroblasts, keratinocytes, endothelial cells, and melanocytes [83]. This specific approach enables the reconstruction of the multilayered structure of human skin with enhanced vascularization and ECM remodeling [83].

## 3. Natural Biomaterials as Bioinks for 3D Bioprinting of Skin Substitutes and Wound-Healing Promoters

The development of complex 3D matrices for wound-healing and skin regeneration using 3D bioprinting requires specialized bioinks. Natural biomaterials used for this purpose are commonly categorized as hydrogels, bioceramics, and carbohydrate- or polysaccharide-based, protein-based, or hybrid-based materials. As such, Table 2 provides a comparative overview of these classes, highlighting differences in printability, mechanical stability, degradation kinetics, and biological performance relevant to skin regeneration and wound-healing.

Natural biomaterials offer excellent biocompatibility and intrinsic bioactivity but are often limited by poor printability, low mechanical strength, variable degradation, and batch-to-batch inconsistency. On the other hand, synthetic biomaterials provide greater mechanical stability, tunable properties, and controlled degradation but generally lack biological cues. However, hybrid biomaterials combine the advantages of both systems by integrating the bioactivity of natural materials with the structural fidelity and printability of synthetic components.

### 3.1. Natural Biomaterials

Natural biomaterials have garnered significant attention for the 3D bioprinting of skin substitutes and wound-healing, primarily due to their inherent biocompatibility, bioactivity, and ability to naturally mimic the extracellular matrix architecture. Natural macromolecules, such as carbohydrate-based biopolymers (e.g., alginate, agarose, cellulose, and chitosan), are derived from biological sources, including animals, plants, and microorganisms. These biomaterials provide an environment that promotes cell adhesion, proliferation, angiogenesis, and re-epithelialization, all of which support skin wound-healing. Alginate, chitosan, and cellulose are among the most widely used natural polymers as hydrogels for wound-healing [84]. Natural polymer-based hydrogels have been extensively studied for wound-healing, as they retain significant water content, naturally mimic the extracellular matrix architecture, and support the encapsulation of a wide range of cell types, including fibroblasts, chondrocytes, hepatocytes, smooth muscle cells, adipocytes, and various stem cells [85]. The biocompatibility of natural hydrogels is attributed to their hydrophilic nature, making them versatile tools for diverse tissue engineering and regenerative medicine applications [86], including 3D printing, biosensors, and wound dressings [87]. Their tunable properties create an optimal environment for cell growth, allowing for precise control over biochemical and biophysical factors that regulate key cellular functions [88].

#### 3.1.1. Carbohydrate- and Polysaccharide-Based Biomaterials

Carbohydrate polymers are commonly selected based on their biocompatibility, versatility, thermo-reversible gelling mechanism, and close resemblance to the human extracellular matrix [89], making them suitable for fabricating functional scaffolds that support skin regeneration and wound repair. Despite their advantages, these biomaterials often lack strong cell adhesion and attachment, requiring chemical modifications. However, such chemical modifications can interfere with the structural integrity of the hydrogel. To enhance the performance and structural integrity of these bioinks, various studies combine carbohydrate-based biomaterials with other biomaterials. For example, carboxymethyl cellulose and Poly-L-lysine hydrogels were modified with glycidyl methacrylate and 3D-printed for wound repair [90]. Similarly, 3D-printed collagen and nanocellulose hybrid scaffolds for skin tissue engineering applications exhibit excellent cytocompatibility and structural fidelity [91]. Carbohydrate-based bioinks are compatible with various bioprinting techniques, including extrusion-based, laser-assisted, and inkjet printing, and have been used to fabricate dermal skin substitutes [92].

Natural carbohydrate-based biomaterials are widely used in 3D bioprinting for the development of skin substitutes and wound-healing applications. Polysaccharide biopolymers, such as chitosan, pectin, and hyaluronic acid, have shown promising applications as bioink materials for wound dressings, drug delivery, scaffolds, and coatings in skin tissue engineering [93]. Polysaccharides like hyaluronic acid (i.e., hyaluronan) are naturally present in the extracellular matrix, offering diverse functions, including structural support, stiffness, tissue regeneration, antimicrobial, antitumor, and immunoregulatory properties [94]. Hyaluronic acid is a component of human connective tissue, while chitosan is derived from arthropod exoskeletons. These biopolymers can be processed into hydrogels and bioinks using advanced methods, enabling their application in regenerative medicine and bioink development [95]. Natural polysaccharides are valued for their bioprinting complex, multilayered tissues due to their diverse chemical structures and tunable mechanical and rheological properties [96]. Additionally, they contain hydrophilic functional groups, such as carboxyl, amino, hydroxyl, and sulfate groups, which facilitate bio-adhesion [97]. However, using polysaccharide-based bioinks for fabricating 3D skin constructs presents several challenges, primarily due to their low mechanical strength and printability [98]. To enhance printability, various bioink synthesis techniques incorporating diverse combinations of natural polymeric materials have been developed [99]. This advances bioink formulations for next-generation 3D bioprinting and wound-healing.

#### 3.1.2. Protein-Based Biomaterials

Protein-based biomaterials, such as collagen, gelatin, fibrin, keratin, and silk fibroin, are cost-effective materials that have garnered significant attention in textiles, food, cosmetics, and biomedical applications, including 3D bioprinting for tissue engineering and wound-healing applications, due to their biocompatibility, low immunogenicity, and native ECM-mimicking features [100]. Natural proteins offer distinct advantages over synthetic proteins (i.e., monobodies or artificial vaccines) as they support cell adhesion, migration, and proliferation, making them well-suited for skin tissue scaffolds [100]. Incorporating proteins into bioinks enhances mechanical strength and toughness, ensures slow degradation, and maintains structural fidelity, while preserving cell viability and tissue integration [101]. For example, a biocompatible pea protein bioink can form complex patterns and maintain optimal cell viability [102].

#### 3.1.3. ECM-Derived Biomaterials

ECM derivatives are emerging as natural sources for biomaterials that possess innate biocompatibility, biodegradability, and low immunogenicity and can propagate cell-specific responses during wound-healing [103]. However, there are still many challenges in clinically translating such ECM-based formulations, primarily due to batch variation, xenogeneic contaminants, and time-consuming and costly processing methods [104,105]. The ECM of native skin is a complex framework of structural and signaling molecules, including collagen, elastin, laminin, fibronectin, proteoglycans, and glycosaminoglycans, that regulate cell adhesion, migration, proliferation, and differentiation during tissue repair [103]. Once tissues are decellularized (i.e., enzymatically, chemically, or physically), the cellular content is removed while maintaining the overall ECM composition and optimal bioactivity. The resulting bioactive scaffold contains native growth factors and biochemical cues that effectively replicate the native skin environment [103]. Different organs and tissues (e.g., the placenta, heart, skin, and adipose tissue) serve as common reservoirs for ECM proteins [106]. ECM-derived hydrogels and powders derived from animal tissues, such as dermis, placenta, and small intestinal submucosa, have demonstrated enhanced angiogenesis, cell infiltration, and re-epithelialization for cutaneous wounds [103,107]. Recent studies have also explored integrating skin-derived decellularized extracellular matrix (dECM) into 3D bioprinting to develop more complex constructs. Skin-derived dECM bioinks retain important ECM components, such as collagen and laminin, promoting keratinocyte adhesion and fibroblast proliferation, which is critical for restoring the epidermis and dermis [106,108].

##### Eggshell Membrane: An Emerging Bioink for Wound-Healing?

Eggshell membrane (ESM) is a natural fibrous biological polymer located between the egg white and calcified eggshell in the avian egg. ESM has been historically described as a dressing material for skin wound-healing in traditional Chinese medicine [109]. ESM has been exploited for various wound-healing applications [110,111,112]; however, ESM remains significantly underutilized despite its useful bioactive properties, natural composition and structure [105,110,111,112]. Rich in collagen, glycosaminoglycans, and antimicrobial peptides, ESM closely mimics the natural extracellular matrix (ECM), promoting cell attachment and tissue regeneration [113,114]. The ECM is a 3D fibrous network that provides structural support to cells but also influences their behavior (proliferation, differentiation, and migration) by providing biochemical and mechanical signaling cues in a gradient fashion [115,116]. These gradient patterns naturally exist within and between tissues, guiding cell migration in the ECM toward the gradually increasing concentrations of soluble signal factors and ECM ligands during the wound-healing process [117]. The avian eggshell membrane contains antimicrobial proteins and peptides, such as lysozyme C, ovotransferrin, ovocalyxin-36, ovocalyxin-32, ovocleidin-17, AvBD-9, and AvBD-10, which provide antibacterial protection through various mechanisms [118,119]. ESM can create an extracellular matrix-like environment, which supports the proliferation of human dermal fibroblasts in vitro [120]. ESM particles accelerated healing in a mouse excisional wound splinting model [121,122], leading to the development of DermaRep™, a processed ESM powder-based wound-healing medical device [122]. More recently, it was shown that particalized ESM (PEM) exhibits enhanced antimicrobial and anti-inflammatory bioactivities in a size-dependent manner [121]. Specifically, PEM particles < 53 μm exhibit increased antimicrobial efficacy against skin-associated pathogens [121]. Furthermore, in vivo mouse studies demonstrated that processed ESM powder stimulates matrix metalloproteinase (MMP) activity at wound edges and keratinocyte cell proliferation, representing a positive impact on the early stages of wound-healing [123].

### 3.2. Synthetic Biomaterials

Various synthetic hydrogels have been developed as bioinks for 3D bioprinting within the domain of skin regeneration and wound-healing due to their tunable mechanical properties, reproducibility, controlled degradation, and functionality [124]. Unlike natural polymers, synthetic polymers can be chemically modified to meet the ideal printability standards essential for personalized constructs. Among the most used synthetic biomaterials for wound-healing are polyethylene glycol, polyglycolic acid, polylactic acid, and polyurethane [125]. Certain synthetic polymers, such as polyesters, are biodegradable and can be more cost-effective than natural polymer biomaterials [126]. However, there are drawbacks associated with using synthetic materials, such as toxicity and biological inactivity. Additionally, synthetic materials exhibit poor cellular interactions, necessitating the use of alternative natural polymers or surface modifications (e.g., incorporation of adhesion peptides) to improve their compatibility with cells. One routine strategy involves combining synthetic and natural polymers to enhance specific desired features [127].

### 3.3. Hybrid Biomaterials

Although polymeric materials are widely utilized in 3D printing due to their many advantages, including low weight and melting points, cost-effectiveness, and flexibility in printing, these materials often exhibit limited mechanical strength and functionality, which can limit their application in skin tissue engineering and regenerative wound care [128]. To address such limitations, various fillers have been incorporated into polymer-based matrices to further enhance their mechanical and functional properties [128]. Hybrid polymers can achieve optimal shear-thinning and viscosity performance, which are crucial for maintaining high shape fidelity during extrusion-based bioprinting [129]. This innovative strategy has led to the development of composite materials that better resemble the biomechanical environment of native human skin. The inclusion of micro- and nanoscale graphene oxide particles or cellulose–gelatin materials has yielded promising results [130]. For example, the addition of cellulose nanocrystals or silk nanofibers to gelatin–chitosan hydrogels has been shown to improve tensile strength and support dermal fibroblast proliferation, making these combinations ideal for cutaneous wound dressings [131]. Furthermore, nanoengineered hydrogels incorporating graphene oxide and hydroxyapatite possess antimicrobial and anti-inflammatory properties, which are crucial for promoting rapid wound closure [132]. As such, nanoengineered biomaterials, such as nanocomposites and nanoengineered hydrogel bioinks, have significant potential for tissue engineering and skin regeneration, making them a smart choice for developing complex scaffolds and skin substitutes.

As such, the significance of these biomaterial classes lies not only in their composition, but also in how their physicochemical and biological properties translate into wound-healing function. Parameters such as printability, viscosity, crosslinking behavior, mechanical stability, degradation kinetics, and intrinsic bioactivity directly affect scaffold fidelity, cell viability, tissue integration, and regenerative performance. Thus, the choice and design of biomaterials are central to determining how effectively bioprinted constructs can support skin regeneration, including re-epithelialization, vascularization, and extracellular matrix remodeling.

## 4. Complementary Strategies Used in Tandem with Bioprinting of Skin Substitutes for Wound-Healing

**Figure 4:**
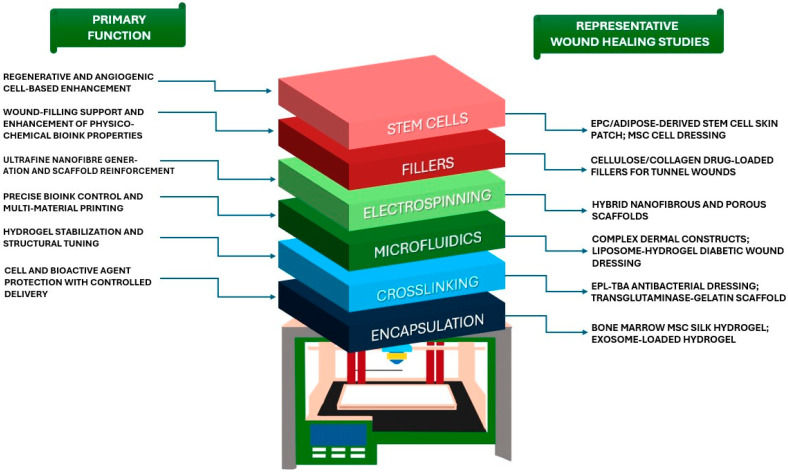
**Summary of complementary strategies used to enhance 3D-bioprinting approaches for skin tissue engineering and wound-healing applications.** This figure overviews the principal strategies discussed in this chapter across three dominant categories: **therapeutics** (**stem cells** and **fillers**) [154,156,157], material design (**encapsulation** and **crosslinking**) [145,150,151,152], and **fabrication technology** (**microfluidics** and **electrospinning**) [137,138,142,143]. For each strategy, its main function and representative wound-healing studies from the cited literature are presented. Together, these approaches enhance the structural, biological, and functional performance of bioprinted skin constructs.

Various material processing methods are used as complementary strategies (Figure 4) to further enhance 3D-bioprinted scaffolds for skin regeneration and wound-healing [133], including electrospinning, microfluidics, laser-assisted techniques, crosslinking, encapsulation, and the use of fillers and stem cells [134]. Since biomaterial properties alone may not fully satisfy the mechanical, biological, and translational demands of skin repair, complementary strategies are often integrated with 3D bioprinting to further enhance scaffold performance, cellular activity, and wound-healing outcomes.

### 4.1. Electrospinning

Electrospinning utilizes high voltage to produce ultrafine polymer fibers, ranging from micrometers to nanometers, with distinct morphologies [135,136]. In this process, a charged polymer solution is dispensed from a spinneret by applying high electric voltage [137]. The polymer jet is then stretched to become thinner and harden before landing on a collector, forming an intricate web of solid fibers [137]. Electrospinning is often used in contrast to additive manufacturing; however, recent studies have shown that integrating the two can help develop porous, nanofibrous scaffolds from polymer solutions that mimic natural extracellular matrices and are used for tissue engineering applications [137,138]. A remaining challenge in processing nanomaterials into polymers is the dispersion of additives and the handling of highly viscous materials [139]. This is where electrospinning can be useful by enhancing the mechanics of 3D-printed materials.

### 4.2. Microfluidics

Microfluidics has emerged as a strong complementary strategy for droplet-based 3D bioprinting, offering precise control over bioink flow and distribution [140]. Integration of microfluidic systems into bioprinting technologies is often referred to as “printhead-on-a-chip,” allowing for rapid, automated selection between multiple bioinks in a single print [141]. This is advantageous for skin tissue engineering applications in order to recreate distinct dermal and epidermal structures. A novel application of microfluidics and coaxial 3D bioprinting technology was used to design liposome–hydrogel dressings, which exhibit enhanced antibacterial activity for diabetic wounds [142]. Similarly, microfluidic 3D printing of a biomimetic polyhydroxyalkanoates-based scaffold promoted skin repair and wound-healing [143]. Overall, microfluidic-based enhancements can yield structurally complex and biocompatible skin substitutes with improved functional integration and potential for enhanced wound-healing.

### 4.3. Crosslinking

Crosslinking is a critical process in 3D bioprinting that stabilizes bioink structures into functional hydrogels by forming a 3D polymer network. This transformation is crucial for maintaining shape fidelity, biocompatibility, and the mechanical integrity of the printed construct [144]. Crosslinking methods are commonly categorized as chemical, physical, or enzymatic, offering different advantages based on the application. Chemical crosslinking forms covalent bonds between functional groups, yielding robust, durable scaffolds suitable for long-term skin applications. A rapidly crosslinking hydrogel (EPL-TBA) using a Schiff base reaction showed improved antibacterial properties as a wound dressing in a rat model [145]. In contrast, physical crosslinking relies on non-covalent or hydrophobic interactions to form reversible, environmentally sensitive hydrogels [146]. Techniques such as ionic gelation and thermal gelation are popular in skin bioprinting due to their tunable degradation and cell-compatible conditions. Sodium alginate/gelatin hydrogels can be crosslinked with calcium chloride to form a nontoxic wound dressing material [147]. Enzymatic crosslinking, on the other hand, uses enzymes as reagents to catalyze the formation of covalent bonds, thereby minimizing side reactions and enabling precise control of hydrogel formation [148,149]. This approach has been successful in crosslinking natural polymers and developing ECM-mimicking scaffolds with tunable physicochemical and self-healing properties [88]. For example, transglutaminase-mediated crosslinking of gelatin can be combined with self-assembled polyelectrolyte nanogels for wound care [150]. The selection of the crosslinking mechanism directly influences the hydrogel’s mechanical and degradation properties, as well as its suitability for skin substitutes and wound-healing applications.

### 4.4. Encapsulation

Three-dimensional bioprinting may also involve the encapsulation of therapeutic cells or bioactive agents within microcarriers typically composed of natural polymers. Encapsulation is the use of biomaterials or biopolymers to secure living cells or drugs. This process enables controlled, localized delivery while maintaining cell viability and minimizing shear stress during printing. Additionally, encapsulation during 3D bioprinting of skin constructs enables controlled delivery of growth factors or drugs. Cell encapsulation creates a living environment that supports cell metabolism. Common biomaterials used for cell encapsulation include natural polymeric materials that are porous, biodegradable, biocompatible, and mechanically robust. Encapsulated bone marrow mesenchymal stem cells in a silk nanofiber hydrogel accelerated wound-healing in a Sprague-Dawley (SD) adult male rat model [151]. Similarly, a biodegradable hydrogel encapsulating human umbilical cord mesenchymal stem cell-derived exosomes promoted collagen deposition and skin regeneration in a female SD rat model [152].

### 4.5. Fillers

Fillers have been incorporated into composite hydrogels to further enhance their physicochemical properties, which affect bioprintability, including shear stress, viscosity, stiffness, elasticity, and surface tension. Inorganic fillers have also been designed to accommodate cell adhesion, proliferation, and differentiation [153]. Polymer-based fillers have been developed to treat challenging wounds, particularly tunnel wounds, which form passages of varying shapes and sizes beneath the skin’s surface. Current wound care treatments primarily target superficial wounds; however, fillers have been incorporated into hydrogel bioinks to mimic the dermal extracellular matrix and restore native skin properties [154]. 3D-bioprinted cellulose/collagen drug-loaded fillers can treat deep tunneling wounds, offering various advantages, including customization, wound exudate absorption, and targeted drug release, to accelerate healing and tissue regeneration [154]. wound-healing products like the 3M™ Tegaderm™ Hydrogel are marketed as fillers to directly promote moist wound environments in dry-to-minimally draining partial and full-thickness dermal ulcers [155].

### 4.6. Stem Cells

The integration of stem cells, particularly mesenchymal stem cells (MSCs), with 3D bioprinting, is now widely employed for skin tissue engineering and wound-healing. Stem cells demonstrate immense potential through their paracrine function, ability to differentiate into skin-specific cell types, and secretion of pro-inflammatory cytokines and growth factors, all of which play a crucial role in facilitating wound repair. In combination with 3D bioprinting, which enables the precise deposition of cells layer by layer, biomimetic skin constructs that mimic the native skin can be fabricated. 3D-bioprinted hydrogels loaded with stem cells are designed to enhance cell viability while promoting angiogenesis and modulating inflammation in full-thickness wound models. For example, a 3D-bioprinted full-thickness skin patch incorporating endothelial progenitor cells and adipose-derived stem cells was reported to accelerate re-epithelialization, neovascularization, and wound closure [156]. Moreover, the integration of MSCs and endothelial cells into a 3D-bioprinted dressing was developed to treat an in vitro model of thermal injury wounds, demonstrating an increase in the expression of wound-healing and neovascularization factors (EGF, PDGF, MMP-9, and TGF-α), as well as a decrease in the expression of the pro-inflammatory factor IL-6 [157]. Despite progress in this field, further work is needed to optimize the proliferative ability of stem cells, ensure cellular viability during printing, and facilitate the functional integration of stem cells with host tissue during wound-healing.

## 5. Future Directions in 3D Bioprinting for Skin Tissue Engineering and Wound-Healing

Looking ahead, advancements in modern Three-dimensional bioprinting are expected to revolutionize biomedical applications by enabling the fabrication of biologically active, in vitro bionic structures [158]. 3D bioprinting has been applied to engineer various tissues across different fields, including bone, skin, and muscle. Next-generation bioprinters enable the development of more complex, multilayered skin tissue models that accurately mimic the epidermal and dermal layers, as well as key skin appendages such as hair follicles, sebaceous glands, and sweat glands [159]. Such constructs hold great potential for more accurate skin grafts in wound-healing applications. A breakthrough has been seen with in situ bioprinting, where bioinks containing specific cells and biomaterials are directly printed onto wound beds [160]. For example, portable, handheld bioprinters have demonstrated early success in treating full-thickness wounds in vivo, enabling real-time tissue reconstruction and reducing the need for extensive skin grafts [161]. Another significant advancement has also been observed in the integration of vascularization and innervation in bioprinted skin constructs [162]. Furthermore, advancements in immune-compatible skin grafts or therapies using patient-derived autologous cells or induced pluripotent stem cells have allowed clinicians to combat immune rejection and reduce post-transplant complications [163]. These innovations could lead to the creation of functional human tissues, organs, and tumor models, offering transformative potential for organ transplantation, understanding disease mechanisms, and regenerative medicine [158]. Despite this progress, several innovations are still needed to develop appropriate technologies and the necessary bioinks for skin regenerative applications. Current studies are prioritizing composite hydrogels, such as GelMA, collagen, and alginate blends, to support cell viability and enhance wound-healing properties. Additionally, crosslinking mechanisms, including light-assisted and enzymatic crosslinking, are being explored to enable the rapid stabilization of 3D-printed constructs [164].

## 6. Market Analysis of 3D Bioprinting and Bioinks for Skin Wound-Healing

**Figure 5:**
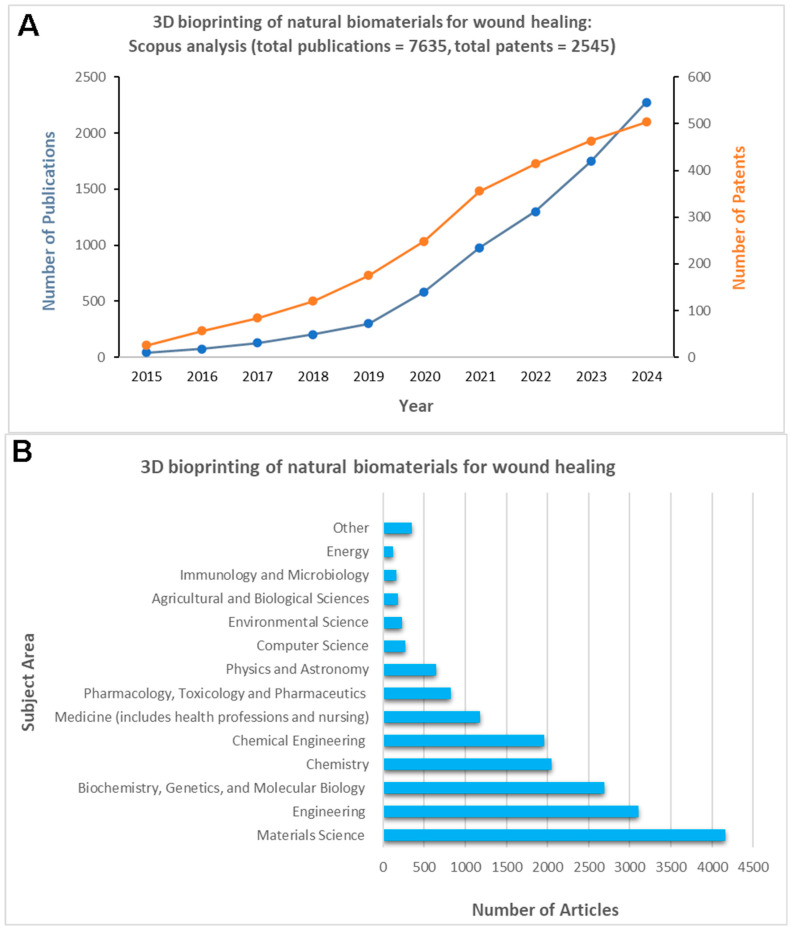

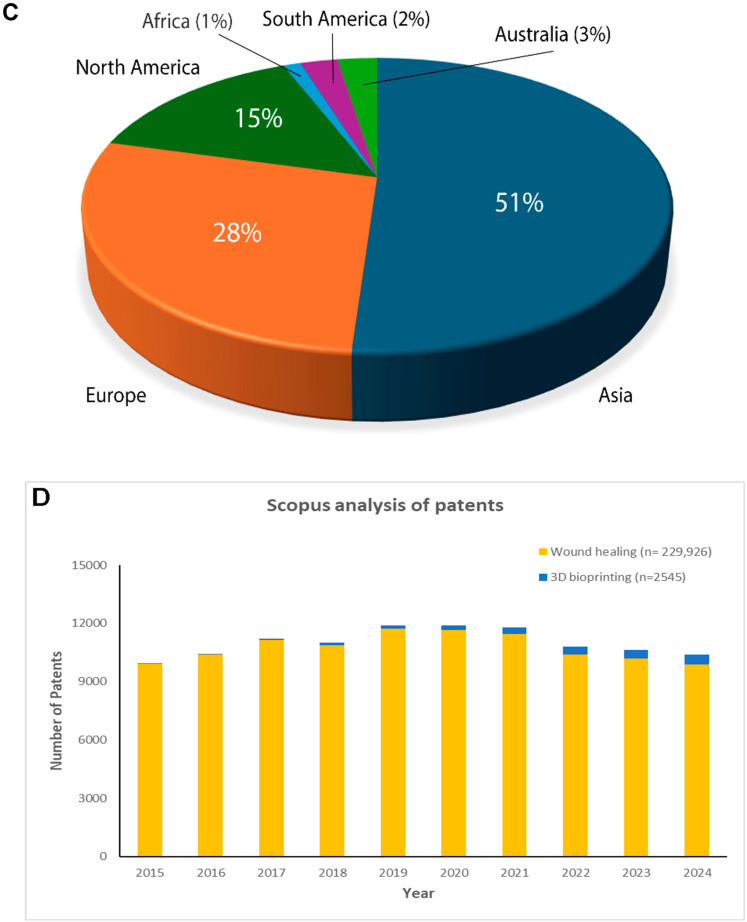
**Progress achieved by the research community in using 3D bioprinting of natural biomaterials for wound**-**healing.** (**A**) Peer-reviewed publications and patents focusing on 3D bioprinting of natural biomaterials for wound-healing applications from 2015 to 2024. The blue line represents the number of peer-reviewed publications, and the orange line represents the number of patents. In Scopus, the search query “3D bioprinting of natural biomaterials for wound-healing” (all fields) was used to identify publications (*n* = 7635) or patents (*n* = 2545) related to the topic. These results were further filtered using the following keywords: hydrogels, bioink, natural materials, and wound repair. (**B**) The number of articles published on this topic in the years 2015–2024, per subject area. (**C**) Geographical distribution of publications by authors’ institute affiliation from 2015 to 2024 pertaining to the relevant topic by continent. (**D**) Number of patent publications on 3D bioprinting (*n* = 2545) and wound-healing (*n* = 229,926) in the period 2015 to 2024. This search was performed using the Scopus database with the keyword search “3D bioprinting and wound-healing” (All fields). Search performed on 27 January 2025.

Over the past decade, the 3D bioprinting of natural biomaterials has been increasingly reported for the fabrication of 3D matrices for wound-healing (Figure 5A), with contributions from researchers across all continents and domains (Figure 5B,C). The publication of patents relevant to wound-healing has remained high overall during the past decade (Figure 5D), with a steady increase in the number of patents related to 3D bioprinting applications (Figure 5A,D). These observations suggest that significant technical breakthroughs are occurring and that researchers are discovering novel methods to leverage 3D printing for biomedical applications, with increased collaboration across different disciplines, such as materials science, engineering, and chemistry [52]. Thus, a growing interest and investment in various industries (e.g., regenerative medicine, food, and pharmaceutical) in further commercializing bioprinting technologies is predicted [18].

### 6.1. Recent Patent and Innovation Landscape of 3D Bioprinting with Natural Biomaterials for Wound-Healing Products

With an increase in patents reflecting clinical translation and technological advancements in this field, the landscape surrounding Three-dimensional bioprinting for wound-healing products has grown dramatically in recent years (>2500 patents over the past 10 years, Figure 5A,D). Significant advancements in 3D bioprinting and bioinks have driven substantial market growth. During the 2015–2024 period, there has been a steady increase in publications and patents that reflect the development of novel materials and the use of 3D bioprinting technologies for wound-healing purposes, further suggesting continued interest and investment from various industries and academic institutions (Figure 5D). Unique and multifunctional bioinks and 3D-bioprinted constructs have been highlighted in recent patents, as indicated in Table 3. These patents are particularly notable, as they have focused on advanced technologies and natural bioink formulations in recent years, enabling more customizable patient-specific products and addressing emergent wound care needs. For example, one novel patent (US20190160203A1) describes a fibrin-RGD-alginate-nanocellulose bioink, optimized particularly for fibroblast adhesion and collagen production, enabling the 3D bioprinting of skin constructs suitable for wound-healing applications [165]. 3D in situ bioprinting strategies also enable the construction of customized, cell-laden planar tissues within wound sites (US11168295B2), allowing for precise spatial control [166]. Moreover, this approach has also led to the development of a methodology, as highlighted in US11564790B2, to construct bioactive tissue layers and skin grafts for wound-healing and skin regeneration [167]. Another recent patent (US11786633B2) describes a 3D-bioprinted in vitro scar tissue model utilizing a specialized bioink composed of polypeptides, cytokines, and fibroblast cells, in combination with extrusion bioprinting, to reproduce natural scar tissue [168]. A step toward biomimetic patient-specific constructs is represented by multilayered bioprinted skin substitutes, such as those described in US11806445B2 [169]. Recent advances in 3D bioprinting (US12115276B2) have enabled the development of collagen-based skin substitutes designed to accelerate wound closure and support skin tissue regeneration [170]. These patented innovations demonstrate the transition from lab-scale prototypes to larger-scale clinical manufacturing, placing 3D bioengineered products at the forefront of the wound care sector.

### 6.2. Market Growth and Technological Advancements in Wound-Healing

The wound-healing market has experienced significant growth worldwide in recent years, primarily driven by an aging population in many developed nations, the rise in vascular diseases and chronic conditions such as diabetes, and an increasing demand from the healthcare industry for effective solutions. This growing demand, along with the substantial economic burden of wound management on healthcare systems, is driving advancements in novel wound-healing technologies and products. As such, the global wound care market was valued at approximately USD 17.49 billion in 2021 and is anticipated to expand to USD 28.23 billion by 2029 [171]. The advanced skin wound care market, which focuses on surgical wounds and chronic ulcers, was valued at USD 11.25 billion in 2024, with a compound annual growth rate (CAGR) of 4.79% from 2025 to 2030 [172]. The increasing number of surgeries related to wound care has driven the high demand for more advanced patient-specific products to ensure efficient healing and prevent further complications. In meeting the growing demand for specialized wound care products, the market is projected to reach USD 14.87 billion by 2030 [172]. The development of state-of-the-art wound care products or technologies, including 3D-bioprinted dressings, is expected to further increase the market share of wound care products. The biomaterials market, particularly in the context of wound-healing products, has experienced significant growth in recent years and is projected to continue rising over the coming decade. Companies like VivoSim Labs, Inc., previously known as Organovo Holdings, Inc., with a market capitalization of USD 6.75 billion, and CELLINK (market capitalization of USD 1.227 billion) have demonstrated substantial growth in the global biomaterial and bioink wound-healing market due to novel formulations and the clinical applicability of products [173,174,175,176].

As of 2024, the global 3D bioprinting market is valued at USD 4 billion and is projected to increase at a CAGR of 17.2% through 2035 [177]. In addition to rapid advancements in 3D bioprinting technology, the growing need for tissue and organ transplants is fueling the expansion of the global bioink market. Investments from industry and academic institutions in bioink research and development are enhancing its use in 3D bioprinting applications for drug discovery, as well as in tissue engineering and regenerative medicine. Bioinks for 3D bioprinting had a market size of USD 224.93 million in 2024, which is expected to grow at a CAGR of 20.48% by 2032, reaching USD 998.57 million [178]. Advancements in regenerative medicine and tissue engineering, particularly in 3D bioprinting, are driving increased demand for bioinks. Tissue-specific bioinks designed for skin tissue engineering models are expected to enhance the effectiveness of bioprinting techniques aimed at replicating native human skin and creating patient-specific constructs, thereby improving chronic wound-healing outcomes. 3D-printed bioinks that incorporate living cells have been shown to promote wound closure and tissue regeneration [179].

Global companies, including Advance Biomatrix (Carlsbad, CA, USA), Rousselot (Son en Breugel, Noord-Brabant, Netherlands), Humabiologics (Phoenix, AZ, USA), and RoosterBi, Inc. (Frederick, MD, USA), are expanding their bioink product lines to meet the growing demand across medical applications, including wound-healing [178]. In 2023, collagen-based bioinks accounted for 19.5% of the bioink market share, making them a preferred material in 3D-printing applications [178]. Additionally, hydrogels accounted for 36% of the market share in 2023, making them a suitable, biocompatible, and biodegradable choice for bioprinting in tissue engineering [178]. There have been significant advancements in 3D-bioprinted hydrogel-based dressings for wound care, which provide a moist environment for healing and protect the wound from contaminants. The adaptability of such dressings has been highlighted by in vitro and in vivo studies, further suggesting their potential in clinical applications [180]. Natural bioinks, such as collagen, chitosan, and gelatin, further dominate the bioink market due to their non-toxicity and biocompatibility [178]. Synthetic polymers currently account for only 10% of the bioink market share; however, with continued research on the horizon, their use is anticipated to increase by 2030 due to the need for tunable mechanical properties similar to those of target tissues [178].

In 2023, medical applications led the 3D-bioprinting market, accounting for a 36.8% share, with a market size valued at USD 2.7 billion in 2026 [181]. The ongoing demand for novel 3D bioprinting techniques in the market, combined with the increasing need for bioengineered organs and tissues, is expected to increase at a CAGR of 23% from 2026 to 2035 [181]. The next 10 years are expected to witness enormous growth in 3D bioprinting, especially in the context of utilizing biomaterials for wound-healing products. This is shown by a global market share increase in USD 8.42 billion and a CAGR of 12.7% by 2035 [182]. Overall trends suggest an upward trajectory of 3D bioprinting in medical applications, including wound-healing. Several companies are focused on integrating 3D bioprinting and bioinks for wound-healing applications. Companies, such as Cellink AB (Gothenburg, Sweden), Allevi Inc. (Philadelphia, PA, USA), and VivoSim Labs, Inc. (San Diego, CA, USA), are actively developing new and improved products to enhance wound care management and treatment using 3D bioprinting [181]. The combination of 3D bioprinting and bioinks offers an incredible approach to wound-healing. However, it is also important to note that the expenses associated with bioprinting equipment, advanced bioinks, and research, as well as regulatory issues, continue to present barriers to the widespread use of 3D bioprinting for tissue engineering worldwide. As the market for 3D bioprinting and bioink use grows, technological advancements and in-kind support from industry and academia are also expected to increase, potentially changing the direction of modern wound care strategies in clinical settings.

#### Market Growth by Geographic Region

Europe and the United States exhibit consistent high demand for advanced wound-healing products, which delineates these regions as dominant global hubs for wound care and drives innovation and commercial growth [183]. The United States leads the 3D bioprinting market, with continued investments in 3D-bioprinted hydrogel-based wound dressings. Furthermore, US-based companies such as CELLINK, VivoSim Labs, and Aspect Biosystems allocate substantial funding to drug discovery and bioprinting research and development initiatives [174,181]. In the United States, chronic wounds affect approximately six million individuals, with yearly treatment costs surpassing USD 25 billion [184]. European countries, notably Germany, France, Spain, and the United Kingdom, are leading research efforts in 3D bioprinting, with a market projected to reach USD 649.91 million by 2028 [185]. In the United Kingdom alone, the National Health Service allocates about £8.3 billion each year for wound care, with £2.7 billion spent on managing healed wounds and £5.6 billion on non-healing wounds [186]. The German 3D-bioprinted human tissue market is expected to grow to USD 113 million by 2035, with a CAGR of 4.8% [185]. This is attributed to a growing demand for tissue regeneration applications and more advancements in precision bioprinting techniques [187]. Across European countries, wound management accounts for 2–4% of total annual healthcare expenditures [188].

The Asia-Pacific wound care market was valued at approximately USD 3.6 billion in 2023 and is projected to increase at a CAGR of 4.8% from 2024 to 2030 [189]. The advanced wound care segment of the market is expected to reach USD 3.31 billion by 2030, with a CAGR of 5.4% by 2030 [189]. In developing countries like India, approximately 3–4% of the diabetic population experience foot-related health issues, taking up 12–15% of the country’s healthcare resources [190]. Southeast Asian countries are also experiencing rising demand for advanced wound care products, with the market valued at USD 221.8 million in 2022 and a CAGR of 6.11% from 2023 to 2030 [191].

### 6.3. Cost/Benefit Analysis for the Clinical Adoption of 3D Bioprinting-Enabled Wound Healing Products

A cost–benefit analysis for the development of wound-healing products using 3D bioprinting and novel bioink formulations is presented in Table 4. As previously discussed, 3D bioprinting enables the customizable development of wound dressings and grafts tailored to individual patient needs and varying wound types (i.e., different shapes and depths), thereby facilitating the ease of integration and functionality of these constructs. Furthermore, with the direct delivery of cells and materials to open wounds, tissue regeneration and wound closure are accelerated. 3D-bioprinted wound dressings and scaffolds can be enhanced to possess antimicrobial, anti-inflammatory, antioxidant, and antitumor properties [192] through the controlled release of bioactive substances and drugs, thereby enabling faster recovery and more effective wound-healing [193]. The localized delivery of antimicrobial drugs can also reduce the risk of infection and minimize complications.

Despite these biomedical benefits, 3D bioprinting for wound-healing also presents several clinical challenges. The long-term performance of these constructs is uncertain due to limited data in this regard. Although various studies have shown promising results for wound closure and reduced scar tissue formation [194], more information is needed on long-term durability and response to mechanical stress [195], as well as the immune-induced responses of 3D-bioprinted wound dressings. Furthermore, combining bioprinted wound dressings with established wound care best practices presents additional challenges for clinicians. The adoption of bioprinting applications in clinical practices necessitates stringent standardization and regulatory frameworks [180]. The use of such technologies will require proper training for professionals in the field.

The initial investment in 3D bioprinting can also be extremely high due to the cost of advanced bioprinter equipment, specialized bioinks and materials, as well as the training required for specialized personnel [180,196]. Despite these financial challenges, 3D bioprinting has the potential to reduce overall, long-term healthcare expenditures by decreasing complications, minimizing additional interventions, and reducing the length of hospital stays and associated costs [180,197]. As bioprinting technologies rapidly evolve and become widespread, the production process is likely to be streamlined for clinical use, making it a more cost-effective option for wound-healing. The integration of 3D bioprinting and bioinks into wound care applications presents significant clinical and economic benefits for major industries (i.e., pharmaceutical, biotechnology, cosmetic, academia, etc.), as seen by successful bioprinting initiatives from companies like Cellink, Organovo, Aspect Biosystems, and 3D Systems [173,181].

### 6.4. Cost-Effectiveness Analysis of Wound-Healing Interventions

The growing clinical demand for advanced wound-healing products has sparked interest in the economic feasibility of 3D-bioprinted skin constructs. Compared to conventional wound dressings, bioprinted scaffolds offer improved customization, bioactivity, and accelerated wound-healing. However, the cost of such products remains a barrier to clinical translation. Commercially available bioengineered skin substitutes, such as Apligraf^®^ (Canton, MA, USA) and Dermagraft^®^ (Canton, MA, USA), have demonstrated improved healing outcomes and reduced hospitalization; however, high production costs limit their widespread adoption [198,199]. Despite this, a review of clinical trial outcomes supports their use for complex, chronic wounds due to reduced complications and lower long-term care costs, as well as the shorter time required to complete wound closure [200,201]. While economic modeling of clinical outcomes for 3D-bioprinted constructs is still in its early stages, early evidence suggests that the rapid fabrication of patient-tailored constructs using natural and synthetic bioinks represents a promising pathway toward improved clinical efficiency and reduced long-term healthcare costs by decreasing infection rates, accelerating healing, and minimizing the need for secondary interventions [200]. Scalable 3D-printing technologies and the development of standardized bioinks will further reduce per-unit costs and the cost per quality-adjusted life year [201]. As healthcare systems advance toward patient-specific care, conducting formal economic evaluations is critical to support regulatory processes and the widespread clinical adoption of bioprinted skin substitutes. To better summarize the clinical and economic advantages of emerging bioprinted solutions, Table 5 presents a comparative analysis of the cost-effectiveness of traditional therapies versus 3D-bioprinted interventions.

### 6.5. Functionality and Clinical Trial Outcomes for 3D-Bioprinted Wound Care Products

The clinical translation of 3D-bioprinted biomaterials for wound-healing has had a significant impact in recent years, particularly in the treatment and management of complex, chronic skin injuries. Recent advancements in extrusion-based 3D bioprinting have led to the development of multifunctional hydrogel constructs that enhance both acute and chronic wound-healing. In the treatment of acute wounds, bioinks such as GelMA infused with platelet-derived growth factor (PDGF-BB) promoted dermal cell attachment, neovascularization, and ECM deposition in vivo [218,219]. In one clinical trial, 3D-printed fibrin hydrogels were extruded onto diabetic foot ulcers, achieving 70% complete healing within 12 weeks without adverse events [220]. Another study also employed extrusion 3D printing to develop a carboxymethyl chitosan and oxidized alginate grafted catechol (0-AlgCat) scaffold, which demonstrated significant antibacterial activity, hemostasis, angiogenesis, and cell proliferation in an infected rat burn model, ultimately reducing the time for wound-healing [221]. Together, these studies highlight the therapeutic potential of 3D bioprinted dressings in enhancing skin regeneration and wound repair.

### 6.6. SWOT Analysis of Technical, Clinical, and Regulatory Factors Influencing 3D Bioprinting and Bioink Development for Wound-Healing

**Figure 6:**
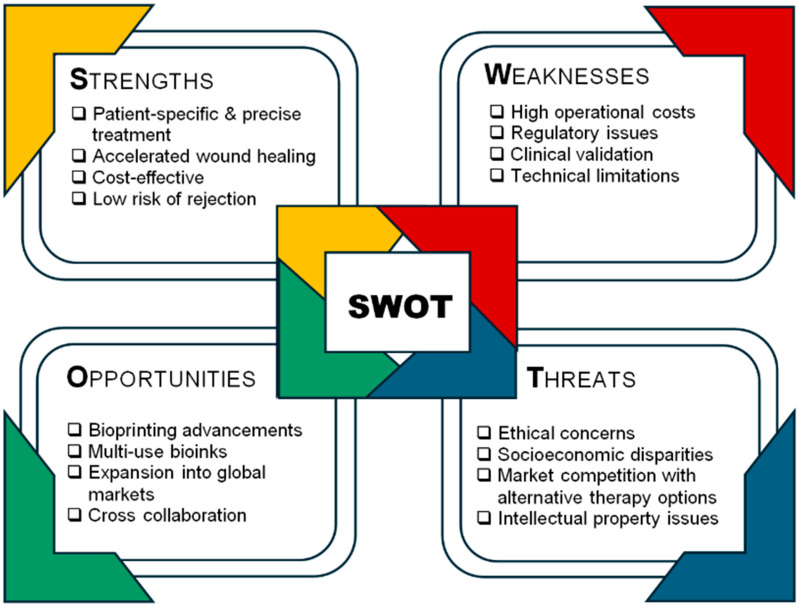
SWOT analysis for the advancement of 3D bioprinting and bioinks for skin wound-healing.

Three-dimensional bioprinting using bioinks represents a great advancement in wound-healing applications, offering more cost-effective and patient-specific treatments, as well as highly effective treatment options. A SWOT analysis assesses strengths, weaknesses, opportunities, and threats, providing a comprehensive framework for business prospects and a deeper understanding of the potential and challenges associated with this novel technology. Various factors may impact the development and marketing of 3D bioprinting and bioinks for wound-healing applications, as highlighted in Figure 6.

Some major strengths include the development of precise, patient-specific tissues, accelerated wound-healing, scalability of applications, cost-effective treatment options, and minimal risk of rejection. Three-dimensional bioprinting enables the development of patient-specific constructs, ultimately improving biocompatibility and reducing the risk of immune rejection through effective tissue integration. More complex structures resembling the natural topography of skin can be formed through the precise layering of cells and biomaterials. Additionally, the addition of growth factors, cells, and natural materials accelerates the wound-healing process and enhances its healing properties.

### 6.7. Limitations and Challenges

However, in addition to these strengths, several drawbacks can limit the advancement of 3D bioprinting and bioinks. One critical issue associated with 3D bioprinting is the high operational costs associated with this technology, which are influenced by bioprinter costs, the development of tissue-specific bioinks, more specialized infrastructure requirements, and maintenance expenses. Commercially available 3D bioprinters range in price from $5000 to $1,000,000 [222]; the cost of advanced bioprinters will increase the initial investment required for different applications and limit accessibility in research and clinical settings. Another major technical challenge is achieving adequate vascularization of bioprinted constructs, which may limit their long-term efficacy and clinical utility. Additionally, variability across bioink formulations, crosslinking strategies, cell-loading conditions, and bioprinting parameters results in inconsistent products and reproducibility issues that diminish clinical validity [223]. In addition, a lack of standardization in bioink composition and printing protocols further limits comparisons across studies and hinders the development of robust, repeatable workflows that are imperative for clinical translation. Furthermore, manufacturing scalability remains a major challenge, as formulations and printing conditions that are optimized at laboratory scale must be adapted for larger-batch production, quality assurance, storage, and distribution [223,224]. Regulatory issues further complicate the potential commercial adoption of 3D bioprinting and bioinks for wound-healing, where classification, risk assessment, quality control, and standardization are stringent processes that must be fully addressed to effectively integrate bioprinted products into clinical practice [224]. Moreover, because many bioprinted wound-healing products combine biomaterials, living cells, bioactive molecules, or patient-specific features, they may not always fit within conventional regulatory categories, further impacting the approval process [224].

The field of 3D bioprinting offers promising opportunities for developing innovative wound-healing products. Current research focused on discovering new bioink combinations, which aims to improve biocompatibility, biomimicry, and mechanical integrity. Incorporating natural polymers can significantly enhance the properties of bioinks and their printing performance [225]. Furthermore, integrating 3D bioprinting with other technologies, such as nanotechnology, can lead to functional scaffolds with improved physicochemical and biological properties [226]. This domain of research also offers opportunities for collaboration among bioprinting companies, research institutions, and industry stakeholders (e.g., pharmaceutical, cosmetic, and regenerative medicine), which can further accelerate the commercialization and advancement of 3D bioprinting for wound-healing applications across global markets.

Despite these opportunities, several other factors pose threats to the progress of 3D bioprinting in wound-healing. The types of human cell lines (i.e., fibroblasts, keratinocytes, and endothelial cells) commonly used for bioinks in the bioprinting process of skin substitutes pose various ethical dilemmas, including donor confidentiality, informed donor consent, potentially invasive cell production procedures, and donor cell ownership [227]. Such ethical concerns surrounding the use of human cell lines and genetic material may lead to public disapproval and regulatory issues [227]. Thus, it is essential to identify and assess the ethical issues associated with novel 3D bioprinting applications before their use in patients [228].

Furthermore, the high cost of 3D-bioprinting equipment and bioinks makes it difficult for this technology to be widely adopted, especially in low-income healthcare settings. Unfortunately, these high costs can limit 3D bioprinting to well-funded institutions and wealthier countries with greater resources. Market competition from alternative wound therapy options, such as stem cell therapy and traditional wound care products (e.g., foams, films, and hydrocolloids), could potentially impact the adoption of novel bioprinting solutions. Additionally, intellectual property issues, such as patents, copyrights, and trademarks, that stem from the rapid development of bioprinting technologies can significantly impact the adoption of 3D bioprinting for wound-healing applications [227].

## 7. Recommendations/Future Perspectives

The advancement of 3D bioprinting for skin wound-healing holds great promise; however, several critical directions need to be prioritized to ensure the effective translation of research into clinical practice. Future efforts should focus on developing standardized, reproducible bioinks that are biocompatible, mechanically stable, and cost-effective. Incorporating natural polymers, smart biomaterials, and nanotechnology into bioink design can further enhance shape fidelity, vascularization, and regenerative properties. Furthermore, stem cell integration into bioinks can provide a unique avenue for creating more personalized and biomimetic constructs that accelerate wound-healing.

It is also equally necessary to address vascularization and innervation concerns in bioprinted skin substitutes. Without sufficient blood supply and neuronal communication, long-term graft survival and functionality are limited. Research should explore hybrid strategies that combine 3D bioprinting with microfluidics, growth factor delivery, and bioactive drugs to promote functional integration with skin tissue. Regulatory frameworks and ethical considerations also need further attention. Developing clear standards for quality control, donor cell sourcing, and product classification will streamline approval processes while ensuring safety and public trust. Continued multidisciplinary collaboration among researchers, clinicians, and industry partners is imperative for advancing the commercialization of novel products. Additionally, strategies to reduce production costs and improve scalability will help make wound therapies more accessible worldwide. Finally, future work should prioritize in vivo validation and clinical trials to evaluate the safety and long-term efficacy of bioprinted products for wound-healing and skin regeneration. Establishing larger-scale biomanufacturing platforms and technologies could have a positive impact on the clinical and advanced wound care markets. By integrating technology with streamlined regulatory, ethical, and clinical processes, 3D bioprinting can transform from a promising laboratory tool into a meaningful solution for global wound care.

## 8. Conclusions

Over the past decade, 3D bioprinting has transformed from a concept into a rapidly evolving biomedical technology with strong clinical and commercial potential for wound-healing. This advancement in the field has been driven by innovations in bioink formulation, the integration of natural and synthetic biomaterials, and improvements in the precision and functionality of bioprinting. Bioprinting enables the customizable design of skin constructs that closely mimic the native extracellular matrix. By incorporating growth factors, stem cells, and smart, stimuli-responsive materials, 3D-printed scaffolds have demonstrated promise in promoting angiogenesis, collagen deposition, and wound closure in both acute and chronic wound models.

Global market analyses have consequently reflected a surge in investment and industrial support for bioprinting technologies, with projections indicating exponential growth in both the wound care and bioink sectors. These trends underscore the growing recognition of bioprinting as a cost-effective, patient-specific approach that can reduce long-term healthcare expenditures by accelerating healing and reducing complications. However, it is essential to acknowledge that various barriers remain, including high operational costs, limited standardization, and stringent regulatory issues, which deter widespread clinical adoption.

## Figures and Tables

**Table 1:** Comparison of different bioprinting technologies for skin regeneration and wound-healing.

Printing Technique	Principle	Advantages	Limitations	Strategies to Overcome Limitations	Applications	References
**Droplet-Based Printing**	Controlled formation of droplets over substrate, often cell-laden	High resolution, limited material use.	Limited mechanical strength and nozzle clogging compromise construct stability and printing outcomes.	Optimized nozzle design, substrate interactions, and viscosity tuning to reduce clogging and improve droplet formation.	Microscale skin patterning, growth factor printing	[53,54]
**Inkjet Printing**	Droplet-based technology using thermal, electrostatic, or piezoelectric pulses to eject droplets of bioink	High speed, low cost, precise control of droplets	Limitation to low-viscosity bioinks limits material selection and shape fidelity, and thermal stress damages cells.	Use low-viscosity and cell-compatible bioinks with controlled temperature and piezoelectric systems.	Skin patterning, gradient biomolecule delivery, epidermal layer deposition	[54,55,56]
**Laser-Assisted Printing**	Droplet-based technology using laser energy to propel droplets of bioink onto a substrate.	High resolution, nozzle-free, minimal shear stress	Complex workflow, expensive parts, time-consuming, and limited scalability and clinical translation	Improve automation, simplify system configuration and optimize laser-transfer parameters for reproducibility and wider adoption	Cell patterning, co-cultures, high-precision skin models	[57,58]
**Extrusion-Based Printing**	Pneumatic, piston, or screw force extrudes bioink through a nozzle	Encapsulated cells, multi-material usage	Lower resolution limits structural patterning, and high shear stress damages cells and reduces viability.	Shear-thinning bioinks; wider nozzle tips, and adjusted pressure and speed parameters	Full-thickness skin constructs, dermal–epidermal scaffolds, wound dressings	[59]
**Fused Deposition Modeling (FDM)**	Thermoplastic filament is melted and deposited layer-by-layer	High mechanical strength, customized scaffolds	Not cell-compatible during printing due to the high temperature from filament melting	Print acellular support frameworks, followed by secondary cell seeding	Outer support structures, hybrid skin constructs with hydrogel core	[60]
**SLA-Based Printing (Stereolithography)**	Photo-polymerization of liquid resin or bioink	High resolution, fast printing	Limited to photo-cross-linkable materials and utilizes photo initiators that may induce cytotoxic effects	Development of biocompatible, photo-crosslinkable bioinks with optimized light exposure for reduced cytotoxicity	Vascularized skin scaffolds, microchannel constructs, and ECM-mimicking geometries	[60,61]

**Table 2:** Comparison of natural biomaterials used as bioinks for skin regeneration and wound-healing.

Biomaterial Type	Examples	Biocompatibility	Printability	Mechanical Strength	Degradability	Cell Adhesion	Special Features	Preferred Wound Context/Key Issues
**Carbohydrate**	Alginate, Agarose, Cellulose, Chitosan, Hyaluronic acid, pectin	High	Good to moderate (depends on blend and crosslinking)	Low to Moderate	Biodegradable (varies with formulation)	Low (often modified)	Resembles ECM; thermo-reversible; antimicrobial and immunoregulatory; chemical modifications improve adhesion and strength.	Preferred for exudative or irregular wounds due to high water absorption, gelation, and ability to conform. Key issues include weak intrinsic cell adhesion and mechanical strength.
**Protein**	Collagen, Gelatin, Fibrin, Keratin, silk fibroin	High	Good	Moderate	Biodegradable (slow)	Excellent	Highly bioactive and low immunogenicity support cell growth, making it a stable, cell-compatible option.	Preferred for regenerative wounds requiring strong cell attachment, migration, and ECM replication. Key issues include weak structural stability, rapid contraction or degradation, and batch-to-batch variability.
**ECM-derived**	Decellularized dermis, small intestinal submucosa, placental ECM, adipose ECM, fibronectin, entactin, laminin, eggshell membrane	High	Good	Low to Moderate	Biodegradable (variable)	High	Naturally mimics ECM; promotes cell adhesion and survival; mitigates foreign body response.	Preferred for chronic or full-thickness wounds where biomimicry is required. Key issues include poor standardization and donor/source variability.
**Hybrid**	Nanocellulose–collagen, polymeric composites	High	Excellent (tunable)	High (enhanced)	Customizable	Customizable	Combines the benefits of different biomaterials, enhanced strength, and performance.	Preferred for complex wounds requiring a balance between printability and mechanical strength. Key issues include formulation complexity, difficulty in optimizing component ratios, and limited information on the best combined materials.
**Bioceramic**	Hydroxyapatite, Calcium phosphate, Bioactive glass	High	Low (as pure ink)	High	Slow	Limited (needs support)	Osteoconductive, antibacterial; often used as fillers in composites due to printing limits	Preferred for wounds involving deeper tissue support or composite scaffolds for added mechanical support. Key issues include poor printability, brittle materials, and a lack of compatibility with soft tissues.

**Table 3:** Recent U.S. patents highlighting the advances in 3D-bioprinting technologies of natural biomaterials for skin regeneration and wound-healing. Source: Google Patents.

Patent Number	Title/Scope	Key Innovation	Application to Wound Healing
**US20190160203A1** (2019)	Preparation and application of fibrin-containing or non-containing RGD-conjugated polysaccharide bioinks for the 3D bioprinting of human skin using novel printing heads [165].	A fibrin-RDG-alginate-nanocellulose bioink, used with coaxial printing nozzles to enhance fibroblast adhesion and collagen production.	High-fidelity 3D bioprinting of functional dermal skin constructs for testing and transplantation.
**US11168295B2** (2021)	Tissue printer [166].	Controlled in situ deposition of cell-laden biopolymers and engineered tissues onto wound sites.	Allows for direct printing of biomaterials and cells onto wound beds to create structured planar tissues.
**US11564790B2** (2023)	Skin printer [167].	Method for 3D bioprinting biological tissue structures and skin graft products based on wound imaging.	On-demand fabrication of patient-specific skin grafts or tissue layers to be applied to wounds for re-epithelialization and skin regeneration.
**US11786633B2** (2023)	3D-bioprinted scar tissue model [168].	Utilizes cell-laden bioinks with polypeptides and cytokines to print stratified scar-like tissue models.	Enables in vitro screening of scar treatments and drug testing, with potential applications in regenerative wound therapies.
**US11806445B2** (2023)	Multi-layer skin substitute products and methods for making and using them [169].	Live, artificial, skin substitute products and methods for wound treatment and compound testing of drug candidates.	Enables tailored grafts for full-thickness skin wounds with rapid coverage and healing.
**US12115276B2** (2024)	Additive manufacturing using recombinant collagen-containing formulations [170].	Presents a 3D-bioprinting method that uses a collagen-based bioink for cell viability and structural integrity.	Production of collagen-based 3D-bioprinted skin substitutes to be applied as grafts to promote wound closure and tissue regeneration.

**Table 4:** Cost–benefit analysis of new wound-healing approaches using 3D bioprinting and bioinks.

Factor	Costs	Benefits
**Economical**	⮚ High initial investments from companies/industry ⮚ Operational/maintenance costs	⮚ Reduced long-term healthcare expenditure ⮚ Streamlined processes for surgical procedures
**Technological**	⮚ Investments in technical assistance and training ⮚ Challenges with standardization	⮚ High-precision and multilayered positioning ⮚ Development of functional and mechanically stable bioinks ⮚ Customization and adaptability
**Societal**	⮚ Ethical concerns with research and development ⮚ Public opinion	⮚ Better quality of life due to superior healing ⮚ Economic growth across major industries ⮚ Accessible treatment options
**Regulatory/** **Logistical**	⮚ Complex and lengthy approval process	⮚ Streamlining bioprinter use ⮚ Improved regulatory frameworks for product approval and market entry
**Clinical** **Outcomes**	⮚ Uncertain long-term effectiveness ⮚ Integration with traditional treatments	⮚ Personalized grafts and constructs ⮚ Accelerated wound-healing and closure. ⮚ Lower risk of infection and complications

**Table 5:** Cost-effectiveness comparison of wound-healing interventions.

Intervention	Estimated Cost	Clinical Benefit	Economic Impact	References
**Conventional dressings**	$10–$50 per application	Suited for superficial, acute wounds	Accessible and low initial cost; however, frequent changes, extended healing times, and increased hospital visits drive long-term expenditures and economic burdens on health systems.	[202,203,204,205]
**Oasis (acellular skin substitute)**	$360–$640 per sheet	Intended for partial and full-thickness wounds, venous ulcers, diabetic ulcers, surgical wounds, etc.	Lower cost alternative for simple, acute wounds.	[206,207,208,209]
**Experimental 3D-bioprinted skin substitutes or dressings**	Estimated $500–$1000 per application	Customizable and bioactive constructs for irregular, deep wounds; accelerated wound-healing with antibacterial effects	High initial costs: However, there is potential to increase production efficiency and reduce healthcare costs through improved outcomes and fewer interventions.	[210,211,212]
**Dermagraft**	$1200–$1800 per application	Human fibroblast-derived dermal skin substitute to stimulate healing in full-thickness diabetic foot ulcers (DFUs), re-epithelialization and tissue regeneration	Although the initial cost is high, patient quality of life is significantly improved in severe cases.	[199,213,214,215]
**Apligraf (cellular skin substitute)**	$1500–$2000 per graft	Enhanced wound-healing (e.g., DFUs and venous leg ulcers); limited need for secondary treatment	Cost-effective for long-term care of acute and chronic wounds.	[198,199,216,217]

## Data Availability

Data sharing is not applicable to this article.
